# Studying plant autophagy: challenges and recommended methodologies

**DOI:** 10.1007/s44307-023-00002-8

**Published:** 2023-10-26

**Authors:** Hua Qi, Yao Wang, Yan Bao, Diane C. Bassham, Liang Chen, Qin-Fang Chen, Suiwen Hou, Inhwan Hwang, Li Huang, Zhibing Lai, Faqiang Li, Yule Liu, Rongliang Qiu, Hao Wang, Pengwei Wang, Qingjun Xie, Yonglun Zeng, Xiaohong Zhuang, Caiji Gao, Liwen Jiang, Shi Xiao

**Affiliations:** 1 Guangdong Laboratory for Lingnan Modern Agriculture, Guangdong Provincial Key Laboratory of Agricultural & Rural Pollution Abatement and Environmental Safety, College of Natural Resources and Environment, South China Agricultural University, Guangzhou, 510642 China; 2 State Key Laboratory of Biocontrol, Guangdong Provincial Key Laboratory of Plant Resources, School of Life Sciences, Sun Yat-Sen University, Guangzhou, 510275 China; 3 School of Agriculture and Biology, Shanghai Jiao Tong University, Shanghai, 200030 China; 4 Department of Genetics, Development and Cell Biology, Iowa State University, Ames, IA 50011 USA; 5 College of Life Sciences, South China Agricultural University, Guangzhou, 510642 China; 6 Key Laboratory of Cell Activities and Stress Adaptations, Ministry of Education, School of Life Sciences, Lanzhou University, Lanzhou, 730000 China; 7 Division of Integrative Biosciences and Biotechnology and Department of Life Sciences, Pohang University of Science and Technology, Pohang, 37673 South Korea; 8 Institute of Plant and Food Science, Key Laboratory of Molecular Design for Plant Cell Factory of Guangdong Higher Education Institutes, Department of Biology, School of Life Sciences, Southern University of Science and Technology (SUSTech), Shenzhen, Guangdong 518055 China; 9 National Key Laboratory of Crop Genetic Improvement, Huazhong Agricultural University, Wuhan, 430070 China; 10 MOE Key Laboratory of Bioinformatics, Center for Plant Biology, School of Life Sciences, Tsinghua University, Beijing, 100084 China; 11 MOE Key Laboratory of Horticultural Plant Biology, College of Horticulture and Forestry Sciences, Huazhong Agricultural University, Wuhan, 430070 China; 12 State Key Laboratory for Conservation and Utilization of Subtropical Agro-Bioresources, Key Laboratory for Enhancing Resource Use Efficiency of Crops in South China, Ministry of Agriculture and Rural Affairs, Guangdong Provincial Key Laboratory of Plant Molecular Breeding, South China Agricultural University, Guangzhou, 510642 China; 13 School of Life Sciences, Centre for Cell & Developmental Biology, State Key Laboratory of Agrobiotechnology, The Chinese University of Hong Kong, New Territories, Shatin Hong Kong, China; 14 Guangdong Provincial Key Laboratory of Biotechnology for Plant Development, School of Life Sciences, South China Normal University, Guangzhou, 510631 China

**Keywords:** ATG8 lipidation, Autophagy, GFP-ATG8 cleavage, Methodology, Microscopy analysis

## Abstract

In plants, autophagy is a conserved process by which intracellular materials, including damaged proteins, aggregates, and entire organelles, are trafficked to the vacuole for degradation, thus maintaining cellular homeostasis. The past few decades have seen extensive research into the core components of the central autophagy machinery and their physiological roles in plant growth and development as well as responses to biotic and abiotic stresses. Moreover, several methods have been established for monitoring autophagic activities in plants, and these have greatly facilitated plant autophagy research. However, some of the methodologies are prone to misuse or misinterpretation, sometimes casting doubt on the reliability of the conclusions being drawn about plant autophagy. Here, we summarize the methods that are widely used for monitoring plant autophagy at the physiological, microscopic, and biochemical levels, including discussions of their advantages and limitations, to provide a guide for studying this important process.

**Supplementary Information:**

The online version contains supplementary material available at 10.1007/s44307-023-00002-8.

## Introduction

Autophagy is an intracellular degradation mechanism that sequesters cytoplasmic components and delivers them to the vacuole or lysosome for breakdown (Zhuang et al. 2015; Michaeli et al. 2016; Marshall and Vierstra 2018). Autophagy is evolutionarily conserved in eukaryotes (Marshall and Vierstra 2018). To date, three forms of autophagy have been described in plants: microautophagy, macroautophagy, and mega-autophagy (Marshall and Vierstra 2018). Macroautophagy (hereafter referred to as autophagy) is the major form of autophagy in plants (Qi et al. 2021). In plants, autophagy is primarily induced by a variety of biotic and abiotic stresses and plays an essential role in maintaining glucose-mediated root meristem activity in Arabidopsis (*Arabidopsis thaliana*) (Huang et al. 2019a). During plant autophagy, autophagic substrates such as aggregated proteins or damaged organelles are surrounded by a double-membrane, open-ended structure termed the phagophore; closure of the phagophore forms a double membrane–bound vesicle termed the autophagosome. Autophagosomes are delivered to the vacuole, where their cargoes are degraded by resident hydrolases (Bassham 2009; Floyd et al. 2012; Li and Vierstra 2012; Zhuang et al. 2015; Michaeli et al. 2016; Marshall and Vierstra 2018; Qi et al. 2021).

The autophagy machinery is composed of a set of autophagy-related (ATG) proteins that are evolutionarily conserved across eukaryotes. Since the first autophagy-related gene, *ATG1*, was identified in 1997, over 40 *ATG*s have been characterized in yeast (*Saccharomyces cerevisiae*) (Matsuura et al. 1997; Fukuda and Kanki 2021). Subsequently, many orthologs of yeast ATGs have been identified in plants (Qi et al. 2021). In plants, these ATGs form several protein complexes, including the ATG1–ATG13 kinase complex, the class III phosphatidylinositol 3‐kinase (PI3K) complex, the ATG9 membrane delivery complex, the ATG8–phosphatidylethanolamine (PE) and ATG5–ATG12 conjugation systems, and the *N*-ethylmaleimide-sensitive factor attachment protein receptor (SNARE) complex. These complexes drive autophagosome formation and fusion with the vacuole membrane (tonoplast) for final degradation in the vacuole (Li and Vierstra 2012).

ATG8 is synthesized as an inactive precursor that is processed by the ATG4 protease to expose a C-terminal glycine residue; this residue is conserved among all family members (Marshall and Vierstra 2018). The resulting mature and activated ATG8 binds to the ATP-dependent E1-activating enzyme ATG7, before being transferred to the E2-conjugating enzyme ATG3 and finally attached to the lipid PE with the aid of an ATG8-specific E3 ligase complex containing ATG5, ATG16, and a second ubiquitin (Ub)-fold protein, ATG12 (Li and Vierstra 2012). In parallel, with the help of ATG7 and the ATG12-specific E2 ATG10, ATG12 is conjugated to ATG5, forming the ATG5–ATG12 conjugation system (Li and Vierstra 2012; Marshall and Vierstra 2018). ATG12 and ATG8 adducts (synthesized *in planta* or in vitro using conjugation reactions) can be reconstituted into liposomes and undergo shifts in electrophoretic mobility that are readily detected by SDS-PAGE followed by immunoblotting with anti-ATG5 or anti-ATG8 antibodies (Thompson, et al. 2005; Phillips et al. 2008; Fujioka et al. 2008; Chung et al. 2010).

The ATG8–PE adduct coats the expanding phagophore and decorates the outer and inner membranes of autophagosomes. Eventually, ATG8–PE adduct on the outer membrane are delipidated by ATG4 and released for reuse, whereas ATG8–PE adduct on the inner membrane are degraded in the vacuole by resident hydrolases (Yoshimoto et al. 2004). Using confocal fluorescence microscopy and fluorescent protein (FP)–ATG8 fusions, autophagosomes within the cytoplasm and autophagic bodies within the vacuole can be detected after stabilization by the H^+^-ATPase inhibitors concanamycin A (ConA) or the cysteine protease inhibitor E64d (Yoshimoto et al. 2004; Contento et al. 2005; Thompson et al. 2005; Izumi et al. 2015; Li et al. 2015).

Genetic analyses in Arabidopsis have shown that most *ATG* knockout or knockdown mutants display premature leaf senescence under normal growth conditions, hypersensitivity to nutrient deficiency (Doelling et al. 2002; Chung et al. 2010), and phenotypes typically associated with impaired autophagy activity. Furthermore, these mutants exhibit altered tolerance to biotic and abiotic stresses and distinct metabolome profiles (Xiong et al. 2007; Hayward et al. 2009; Liu et al. 2009; Guiboileau et al. 2012; Avin‐Wittenberg et al. 2015; Chen et al. 2015; Qi et al. 2021).

The regulatory network controlling autophagy has been elucidated through the development of autophagy detection technologies. Increasing evidence has demonstrated that during autophagosome formation in plants, the activities and stabilities of ATG proteins are strongly affected by posttranslational modifications, particularly phosphorylation, ubiquitination and persulfidation. The kinases TARGET OF RAPAMYCIN (TOR) and SUCROSE NON-FERMENTING 1-RELATED KINASE 1 (SnRK1) play negative and positive roles, respectively, in regulating autophagy, possibly by modulating the stability and activity of the ATG1–ATG13 kinase complex and the core component of the PI3K complex (Liu and Bassham 2010; Chen et al. 2017a; Pu et al. 2017; Huang et al. 2019b). Furthermore, recent studies have demonstrated that members of the RING-type E3 Ub ligase protein family SEVEN IN ABSENTIA OF *ARABIDOPSIS THALIANA* (SINAT) differentially regulate the stability of ATG1–ATG13 and ATG6 by modulating their proteolysis, thus helping regulate autophagy (Qi et al. 2017; 2020; 2022a). More recently, two 14–3-3 adaptors, 14–3-3λ and 14–3-3κ, which specifically associate with phosphorylated ATG13s, were shown to modulate ATG1–ATG13 complex formation and facilitate SINAT-mediated proteolysis of ATG13s, thus redundantly modulating autophagy dynamics (Qi et al. 2022b). Moreover, the signaling molecule hydrogen sulfide regulates essential processes in plants, such as autophagy. In Arabidopsis, persulfidation of ATG4 and ATG18a is involved in autophagy in response to environment cues, such as treatment with abscisic acid or agents that induce endoplasmic reticulum (ER) stress (Laureano-Marín et al. 2020; Aroca et al. 2021).

Major advances have recently been made in revealing the molecular and functional mechanisms of autophagy in plants using molecular genetic, cell biological, and biochemical approaches (Bassham 2015; Chen et al. 2017b; Marion et al. 2018). Several methods have been developed to detect autophagy in plant cells, including phenotypic studies to analyze tolerance to nitrogen or fixed-carbon starvation and onset of leaf senescence under normal growth conditions; cell biological methods to detect autophagosome formation; and biochemical methods to detect the accumulation of ATGs.

However, some of the methodologies are prone to misuse or misinterpretation, which may cast doubt on the reliability of the conclusions being drawn about plant autophagy. In this review, we summarize techniques useful for detecting and quantifying autophagy in plants, discuss their advantages and limitations, and highlight the considerations needed to ensure that researchers draw appropriate conclusions from these methods.

## Advantages and disadvantages of methods to study autophagy in plants

### Phenotypic analysis

In plants, leaf senescence remobilizes nutrients from senescing tissues to young organs under normal growth conditions and recycles nutrients to support plant survival in response to nutrient limitation (Masclaux-Daubresse 2016). During natural leaf senescence, autophagy is induced and functions as a housekeeping mechanism to degrade intracellular contents and remobilize nutrients (Ishida et al. 2008; Wada et al. 2009; Izumi et al. 2017). Catabolic reactions that occur during leaf senescence are likely to target chloroplasts for degradation, as chloroplasts are dismantled in the early phase of senescence (Avila-Ospina et al. 2014). Under nutrient-replete conditions, most autophagy-defective mutants display few differences from wild type in seed germination, cotyledon development, root elongation, and seed production (Hanaoka et al. 2002). However, *atg* mutants have fewer rosette leaves and display early bolting and premature senescence under long-day (16-h light/8-h dark) or short-day (8-h light/16-h dark) photoperiods (Doelling et al. 2002; Hanaoka et al. 2002; Chung et al. 2010; Li et al. 2014). Early leaf senescence was confirmed by lower chlorophyll content and elevated expression levels of senescence-related genes, such as *SENESCENCE 1* (*SEN1*) and *YELLOW STRIPE LIKE 4* (*YSL4*) (Doelling et al. 2002). These results suggest that autophagy is required to maintain cellular viability and efficient nutrient use throughout the entire plant life cycle.

**Fig. 1:**
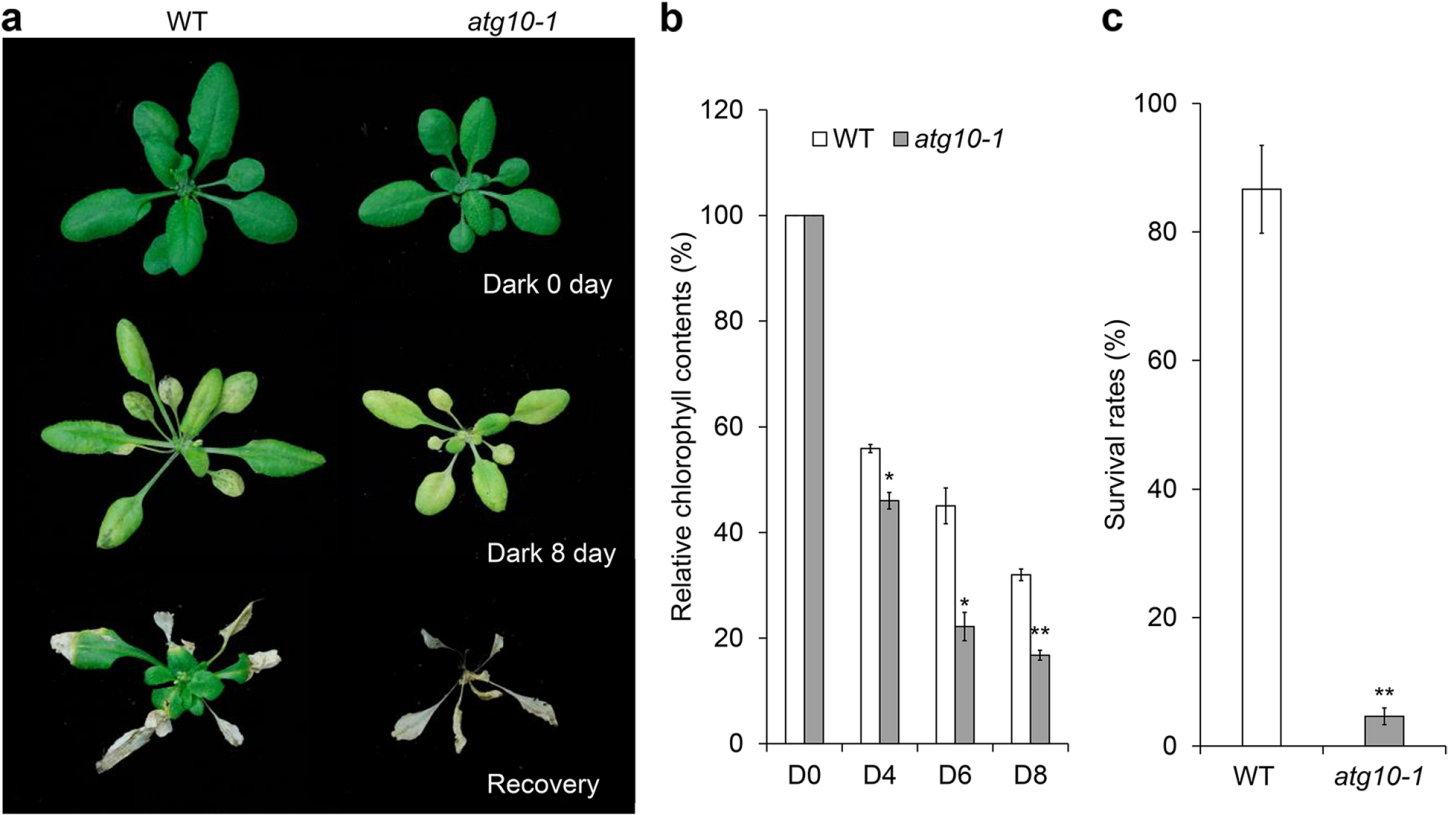
Soil-grown *atg10-1* mutant plants show increased sensitivity to carbon starvation. (**a**) Sensitivity of soil-grown wild-type (WT) and *atg10-1* plants to carbon starvation. Three-week-old plants were grown in normal light/dark conditions (Dark 0 day), followed by growth in constant darkness for 8 d (Dark 8 day). The plants were then allowed to recover under normal light/dark conditions for 7 d (Recovery) before photographs were taken. (**b**) and (**c**) Relative chlorophyll contents (**b**) and survival rates (**c**) of plants after 8-d carbon starvation treatment. Asterisks indicate significant differences from WT (*P< 0.05; **P < 0.01 by Student’s *t* test)

Under nutrient-limited conditions, autophagy contributes to the recycling of damaged or unwanted materials and organelles, including entire chloroplasts, to replenish essential nutrients and generate sufficient resources for the cell to support its vital needs and survival (Masclaux-Daubresse et al. 2017; Qi et al. 2021; Yang et al. 2020a, b). During fixed-carbon starvation, autophagy is involved in vesicular trafficking and degradation of the chloroplast stroma and chloroplast proteins through distinct pathways (Ishida et al. 2008; Michaeli et al. 2014; Izumi et al. 2015). The breakdown of insoluble starch granules in the chloroplast is a key step in respiratory energy production at night in plants, as it generates soluble sugars in leaf mesophyll cells (Smith and Stitt 2007). A study using *Nicotiana benthamiana* leaves revealed the contribution of autophagy to starch granule degradation at night (Wang et al. 2013). Overall, the degradation of chloroplast components by autophagy is likely to be induced in response to sugar starvation. The deletion of *ATG* genes in Arabidopsis disrupted the normal development of autophagosomes, leading to hypersensitivity to fixed-carbon starvation (Doelling et al. 2002; Hanaoka et al. 2002; Thompson et al. 2005; Xiong et al. 2005; Suttangkakul et al. 2011; Li et al. 2014; Qi et al. 2021; Li et al. 2022). When transferred to darkness for several days and allowed to recover under normal growth conditions, soil-grown *atg* mutants displayed strong sensitivity to the dark treatment, as evidenced by their lower chlorophyll contents and survival rates (Hanaoka et al. 2002; Thompson et al. 2005; Chung et al. 2010; Qi et al. 2017; Fig. 1).

Because the fixed-carbon starvation assay using soil-grown plants is time-consuming, researchers have devised a rapid and robust assay for sensitivity to fixed-carbon limitation using young seedlings grown on agar medium in Petri plates (Chung et al. 2010). Seedlings are transferred to Murashige and Skoog (MS) agar medium (without added sucrose) and incubated in continuous dark conditions for several days until they show poor growth. After recovery under normal growth conditions (in the light), *atg* mutants exhibit decreased chlorophyll contents and survival rates compared to wild-type seedlings (Chung et al. 2010; Suttangkakul et al. 2011; Li et al. 2014; Qi et al. 2017). Thus, carbon starvation treatment of young seedlings grown on agar medium and adult plants grown on soil are both suitable methods for analyzing tolerance of fixed-carbon limitation.

**Fig. 2:**
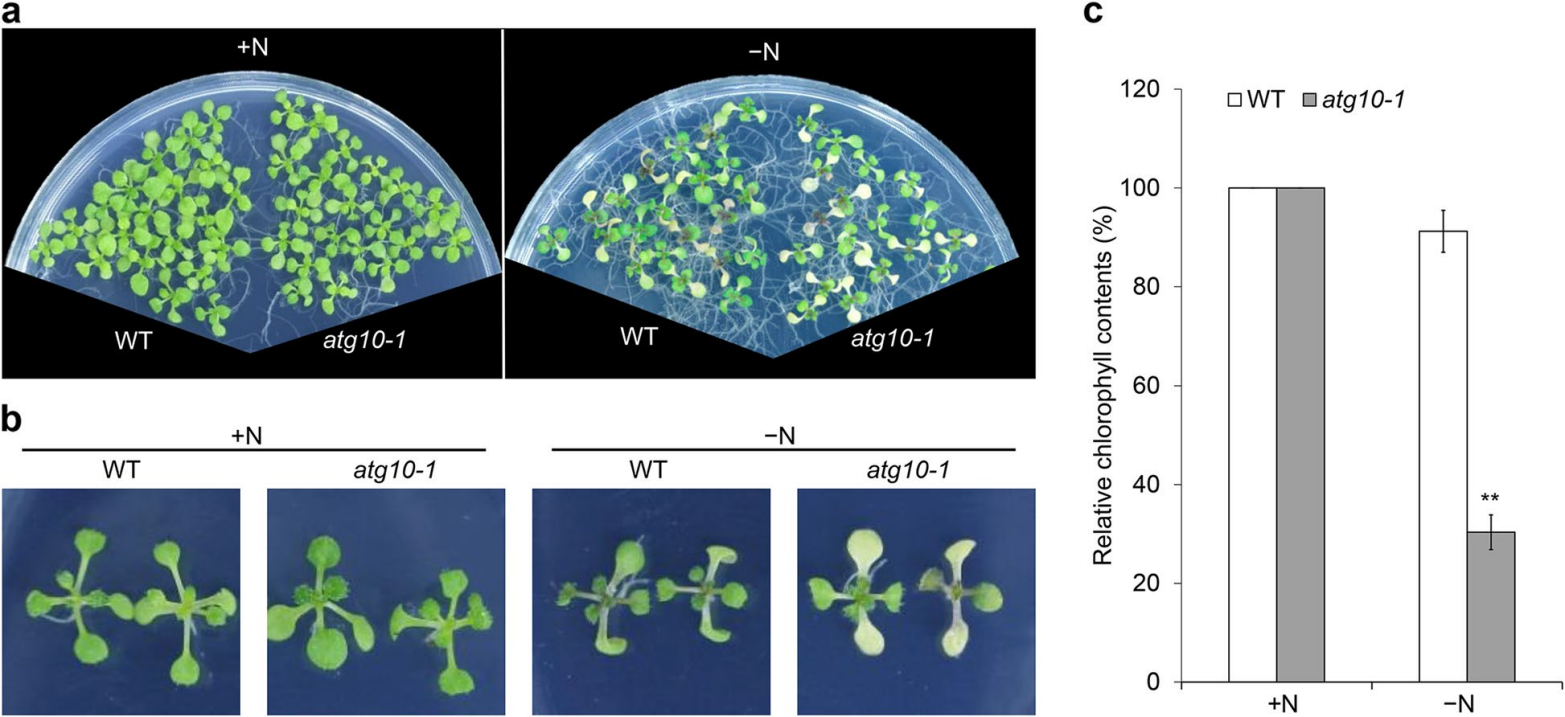
*atg10-1* mutants show decreased tolerance to nitrogen starvation. (**a**) and (**b**) Phenotypes of wild type (WT) and *atg10-1* in response to nitrogen limitation. One-week-old seedlings grown on MS medium were transferred to N-rich (+ N) or N-free (–N) agar medium and photographed 5 days later. (**c**) Relative chlorophyll contents of WT and *atg10-1* seedlings grown under N + or N– conditions shown in (**a**) and (**b**). Chlorophyll contents under N + conditions were set to 100%. ( **P < 0.01 by Student’s *t* test)

Nitrogen (N) is one of the most important nutrients required for plant growth. To cope with nitrogen limitation, plants recycle the N contained in mesophyll cell chloroplasts via chloroplast degradation pathways that partially rely on autophagy (Ren et al. 2014; Masclaux-Daubresse et al. 2017). Although both fixed-carbon and nitrogen starvation result in autophagy-mediated degradation of chloroplasts, the induction of autophagy in response to these conditions likely occurs via distinct mechanisms. Compared to wild-type plants, Arabidopsis *atg* mutants display increased chlorosis and lower chlorophyll content during N limitation (Doelling et al. 2002; Hanaoka et al. 2002; Thompson et al. 2005; Xiong et al. 2005; Qi et al. 2021; Fig. 2). Nevertheless, *atg* mutants accumulate more anthocyanins in response to N starvation under long-day photoperiods, a response not seen in carbon-starved plants (Xiong et al. 2005; Qi et al. 2017). TUMOR NECROSIS FACTOR RECEPTOR-ASSOCIATED FACTOR 1a (TRAF1a) and TRAF1b are adaptor proteins that help regulate autophagy by modulating ATG6 and ATG1 − ATG13 ubiquitination and degradation in plants (Qi et al. 2017; 2020). The loss of TRAF1a and TRAF1b function results in decreased autophagosome formation under nutrient starvation conditions (Qi et al. 2017). Indeed, *traf1a traf1b* double mutants, similar to the canonical autophagy-deficient mutant *atg10-1*, exhibited greater sensitivity to both fixed-carbon and N starvation compared to wild-type plants (Qi et al. 2017).

Thus, autophagy plays an essential role in nutrient recycling during natural senescence and in response to nutrient limitation. Plant phenotypes may serve as a guide for specifying the function of a protein in autophagy. Observation of the leaf senescence phenotype and calculation of survival rates and chlorophyll contents under nutrient-rich and nutrient-limited conditions is a significant and simple physiological method to evaluate autophagy activity in plants. Of course, these phenotypic assays should be complemented and confirmed with more direct measurements of autophagy before drawing conclusions about specific gene function.

### Microscopy analyses

#### Confocal microscopy analyses of *GFP-ATG8e* transgenic plants

Fluorescence microscopy, which detects the abundance of ATG proteins fused to a fluorescent protein, is a reliable method for investigating autophagosome dynamics in plant cells (Pu and Bassham 2016). Nine isoforms of the ATG8 protein are encoded by the Arabidopsis genome (ATG8a–ATG8i) and function partially redundantly in modulating autophagic activity (Doelling et al. 2002; Sláviková et al. 2005). Upon induction of autophagy, the ATG8 precursor is processed by the ATG4 protease to expose a C-terminal glycine residue and is covalently modified with the attachment of the membrane lipid PE via ubiquitin-like reactions that promote the formation of the ATG8–PE adduct (Ohsumi 2001; Yoshimoto et al. 2004; Hanada et al. 2007; Fujita et al. 2008). Although ATG8–PE decorates both the inner and outer membranes of the autophagosome, ATG8–PE on the outer membrane can be removed by cleavage mediated by the ATG4 protease during autophagy. After fusion with the vacuole, inner membrane-associated ATG8 enters the vacuole along with the single-membrane autophagic body and is finally degraded by resident hydrolases (Li and Vierstra 2012; Slobodkin and Elazar 2013). These properties make ATG8 a useful marker to monitor autophagosomes and autophagic bodies in plant cells.

**Fig. 3:**
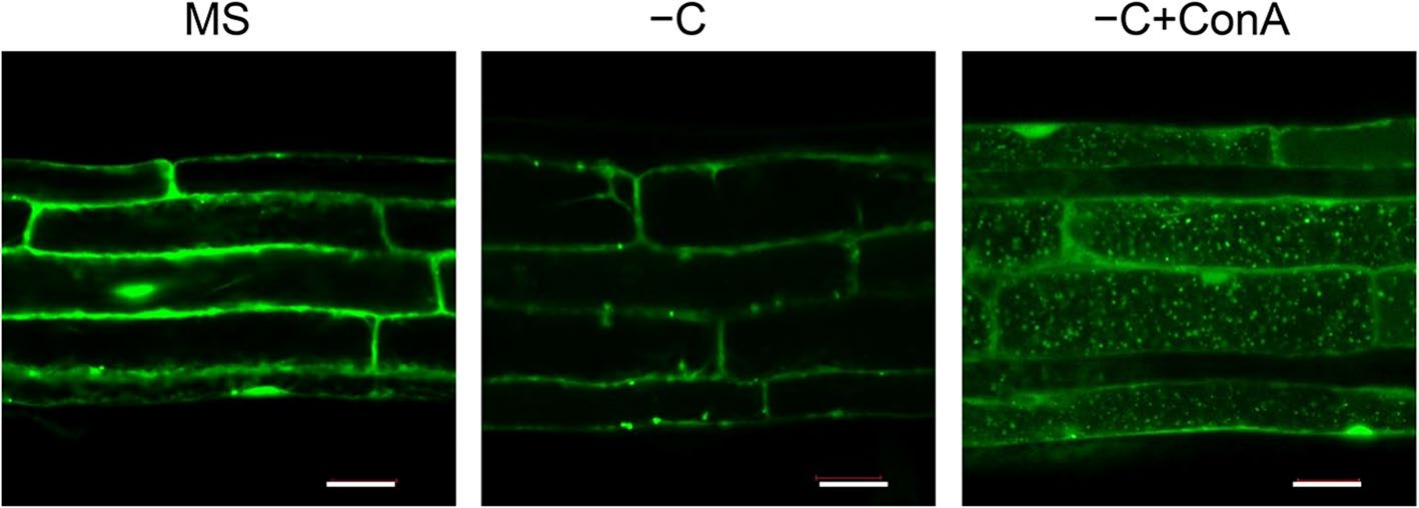
Detection of autophagosomes by confocal microscopy in Arabidopsis root cells. Confocal microscopy of *GFP-ATG8e* transgenic seedlings upon carbon starvation. One-week-old *GFP-ATG8e* seedlings were transferred to MS medium (MS) or sucrose-free liquid MS medium alone (–C) or with concanamycin A (–C + ConA) for 16 h and visualized by fluorescence confocal microscopy. Scale bars, 50 μm

A fusion between the green fluorescent protein (GFP) and ATG8 (GFP-ATG8) is commonly used for visualizing autophagosomes and autophagic bodies in plants (Li and Vierstra 2012; Liu and Bassham 2012). It is worth noting that ATG8 is conjugated to PE at its C terminus before becoming anchored to the phagophore membranes, such that fluorescent proteins can only be added to the N terminus of ATG8 (Yoshimoto et al. 2004; Woo et al. 2014). The construct *GFP-ATG8* encoding the fusion protein can be stably or transiently introduced into Arabidopsis cells and the fusion protein can be detected using confocal microscopy (Yoshimoto et al. 2004; Contento et al. 2005; Thompson et al. 2005; Xiong et al. 2007; Klionsky et al. 2016). In Arabidopsis root cells under normal growth conditions, most of the GFP-ATG8 fusion protein is located in the cytoplasm and is visible as a diffuse fluorescence signal, with a few punctate autophagosomes moving via cytosolic streaming (Fig. 3). A low level of autophagy occurs constitutively in the meristematic zone of Arabidopsis root tips or as basal autophagy for housekeeping (Inoue et al. 2006; Chung et al. 2010; Huang et al. 2019a). Increasing evidence suggests that the formation of GFP-ATG8-labeled punctate structures in plants is rapidly induced by nutrient starvation and several biotic and abiotic stresses in plants (Liu et al. 2018; Qi et al. 2021).

It is important to note that, due to the immediate degradation of autophagic bodies by acid hydrolysis in vacuoles, it is essential to use the inhibitor ConA, a vacuolar H^+^-ATPase inhibitor that prevents vacuole-mediated degradation of intracellular substances by disrupting vacuolar acidification and vesicle trafficking, to assess autophagic flux in plants via confocal microscopy (Matsuoka et al. 1997; Dettmer et al. 2006). Indeed, autophagic bodies that accumulate in the vacuole are easily observed as 1–2-μm vacuolar puncta when stabilized by ConA treatment (Marshall and Vierstra 2018; Fig. 3).

A quantitative assessment of autophagy activity can be obtained by counting the number of GFP-ATG8-labeled autophagosomes in each microscopy frame and calculating their average number across all images for a given genotype or treatment. The average number of autophagosomes in each image indicates the extent of autophagy (Yoshimoto et al. 2004; Contento et al. 2005; Thompson et al. 2005; Izumi et al. 2015; Li et al. 2015). It should be noted that GFP-ATG8 forms punctate protein aggregates when accumulating to high levels; however, these aggregates are typically unevenly shaped rather than spherical (Bassham 2015). In summary, under normal growth conditions, most GFP-ATG8 signal is diffuse throughout the cytoplasm (Fig. 3). After transfer to nutrient limitation conditions, some GFP-ATG8-labeled punctate structures are present mainly in the cytosol due to their immediate degradation in the vacuoles, but accumulate in the vacuole as autophagic bodies upon incubation with ConA (Fig. 3).

#### Other fluorescently tagged proteins for measuring plant autophagy

In addition to ATG8, other autophagy-related proteins also localize to autophagosomes and autophagy-related structures. Fusions of ATG1, ATG13, ATG11, ATG6, or ATG14 to fluorescent proteins localize to punctate structures in the cytoplasm during nutrient limitation conditions, and these are delivered to and accumulate in the vacuole upon application of ConA (Fujiki et al. 2007; Suttangkakul et al. 2011; Li et al. 2014). A study in Arabidopsis suspension cells showed that cyan fluorescent protein (CFP)-tagged ATG6 and yellow fluorescent protein (YFP)-ATG8 exhibited clear enrichment in the punctate structures within the cytoplasm. The fluorescent signals colocalized in bright foci when the cells were incubated in sucrose-free medium (Fujiki et al. 2007), suggesting the colocalization of ATG6 with ATG8. Although the intracellular distribution of ATG6 clearly differs from the Golgi, *trans*-Golgi network (TGN), and endoplasmic reticulum (ER) in Arabidopsis suspension cells (Fujiki et al. 2007), it is worth noting that ATG6 plays an important role in other trafficking systems in addition to autophagy (Harrison-Lowe and Olsen 2008; Patel and Dinesh-Kumar 2008).

When transiently expressed in Arabidopsis leaf protoplasts, GFP-tagged ATG1a and ATG13a usually appear to be confined to the cytoplasm, accumulating within a few puncta that are similar in size to the GFP-ATG8 labeled punctate structures (Suttangkakul et al. 2011). However, testing whether ATG1a and ATG13a colocalize with ATG8a is necessary to verify that ATG1a and ATG13a label autophagosomes or autophagic bodies. ATG11, a core component of the ATG1 − ATG13 complex, helps link the ATG1 − ATG13 complex to autophagic membranes (Li et al. 2014). GFP-ATG11 colocalizes and associates with mCherry-ATG8a in punctate structures in the vacuole of root cells from stable transgenic Arabidopsis lines upon N starvation and ConA treatment (Li et al. 2014). These results indicate that these autophagosomes and autophagic body-bound proteins can be used as autophagosome markers as alternatives to GFP-ATG8.

Recent work showed that a newly defined key component of PI3K, ATG14, is involved in autophagic body accumulation and cargo delivery during nutrient stress (Liu et al. 2020). Confocal fluorescence microscopy analysis of *GFP-ATG14b* transgenic Arabidopsis roots showed that GFP-ATG14 labelled punctate structures colocalized with mCherry-ATG8a reporter and accumulated in the vacuole upon ConA treatment in response to nitrogen starvation, indicating that ATG14 translocated to autophagic bodies under nutrient starvation conditions (Liu et al. 2020). Moreover, fusion of *Nicotiana benthamiana* ATG14 with YFP generated punctate signals that overlapped with CFP-ATG8–labelled autophagic structures and CFP-ATG6–labelled fluorescent dots (Wang et al. 2022a, b), indicating that ATG14 is also an ideal marker for autophagy analysis. Furthermore, ULTRAVIOLET RESISTANCE-ASSOCIATED GENE (UVRAG), a subunit of the PI3K complexes in *Nicotiana benthamiana* colocalized with ATG6 and ATG14a, partially in presence of ATG8-postive autophagic structures (Wang et al. 2022a, b), suggesting it may be useful for autophagy analysis in plants.

In contrast to the potential autophagy markers listed above, ATG5-GFP fluorescence changes from a diffuse signal in the cytosol under normal conditions to punctate and ring-like cytosolic structures that partially colocalize with ATG8 during nutrient starvation, with a portion localizing to the edge of growing phagophores (Le Bars et al. 2014). However, the ATG5-decorated phagophores ultimately dissociate from the mature autophagosome, preventing their delivery to the vacuole (Le Bars et al. 2014). This feature makes ATG5 suitable for distinguishing between intermediates of autophagosome formation and mature autophagosomes. The Bin-Amphiphysin-Rvs domain–containing protein SH3 DOMAIN-CONTAINING PROTEIN2 (SH3P2) colocalizes with ATG6 and ATG9, and translocates to the phagophore during autophagy induction. SH3P2 may facilitate membrane expansion or maturation and mediate autophagy by associating with the PI3K complex and ATG8 during autophagy (Zhuang et al. 2013). These results indicate that, similar to ATG5, SH3P2 would be useful as a marker for the early stages of autophagosome formation. Thus, the coordinated use of markers of autophagy and its intermediates, such as ATG8, ATG5, and SH3P2, fused to fluorescent proteins, may allow us to observe autophagy from its initiation in the cytoplasm to the degradation of target proteins and organelles inside the vacuole.

#### Examination of autophagy by transmission electron microscopy (TEM) in plants

Ultrastructural analysis revealed the general morphology of autophagic intermediates during autophagy in Arabidopsis, showing that double membrane–bound autophagosomes accumulate in the cytoplasm in response to nutrient starvation (Rose et al. 2006). Transmission electron microscopy (TEM) is an outstanding method for analyzing autophagy progression and autophagic structures, such as nucleation and elongation of the phagophore, closure to form double membrane–bound autophagosomes, and release of single-membraned autophagic bodies into the vacuole after fusion of the autophagosome with the tonoplast (Zhuang et al. 2013; 2017; Gao et al. 2015; Zheng et al. 2018). TEM is also the only tool that reveals the morphology of autophagic structures at nanometer resolution (Klionsky et al. 2016; Zhou et al. 2023).

**Fig. 4:**
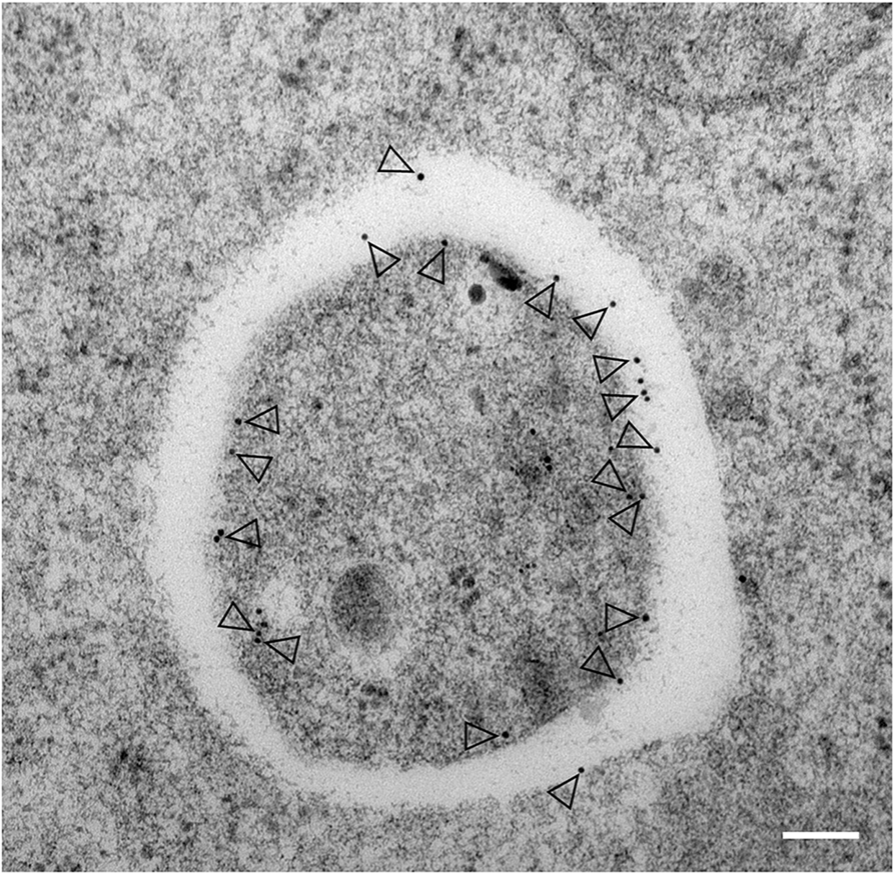
Detection of autophagosomes by transmission electron microscopy in Arabidopsis root cells. Image showing YFP-ATG8e–positive structures immunolabeled with anti-GFP antibodies. A representative TEM image of root cells from *YFP-ATG8e* transgenic seedlings treated with 100 μM BTH for 6 h, followed by immunogold labeling using anti-GFP antibodies. Arrowheads indicate the gold particles for the antibody against ATG8e (10 nm). Scale bar, 100 nm

Under TEM observation, autophagosomes are distinctly visible as double membrane–bound spheroidal structures containing their cargoes targeted for degradation. Nonselective autophagy involves autophagosomes that are 0.5 − 1.5 μm in diameter (Kliosnky et al. 2016; Zheng et al. 2018; Fig. 4), but selective autophagy involves autophagic structures of a wide range of sizes, depending on their specific substrates. In the past few years, TEM observations have revealed that protein aggregates, as well as damaged organelles such as chloroplasts (chlorophagy), peroxisomes (pexophagy), mitochondria (mitophagy), ER (ER-phagy), ribosomes (ribophagy), 26S proteasomes (proteaphagy), and pathogens, can be degraded via autophagy in plants under various physiological conditions (Ran et al. 2020; Luong et al. 2022). Thus, in addition to illustrating the morphology of autophagic structures, TEM can be used to identify the autophagosome cargo targeted for degradation.

The combination of TEM and immunogold labeling, by which target molecules are specifically recognized by primary antibodies bound to gold particles of different sizes, has been widely used in cell biology (Richardson et al. 2022). Immuno-TEM with gold-labeling using antibodies against autophagy-related proteins, such as SH3P2 and ATG8, has been used to label autophagic structures and thereby provide high-resolution spatial information on autophagosome formation (Zhuang et al. 2013; 2017; Gao et al. 2015; Fig. 4). ATG8 immunogold labeling also makes it possible to detect previously undescribed degradative organelles within autophagic compartments. A variety of functional proteins were shown to play important roles in regulating autophagosome formation in plants following the application of this technique (Zhuang et al. 2013; 2017; Gao et al. 2015).

To elucidate the ultrastructure of SH3P2-GFP-positive compartments using immunogold-EM, *SH3P2-GFP* transgenic Arabidopsis plants were first subjected to BTH [benzo-(1,2,3)-thiadiazole-7-carbothioic acid *S*-methyl ester] treatment to induce autophagy, followed by sample fixation via high-pressure freezing/freeze substitution and subsequent immunogold labeling using anti-SH3P2 antibodies. Immunogold-EM observations showed that the diameters of SH3P2-positive structures range from 300–1000 nm and that SH3P2 predominantly localized to the membrane surface and was involved in membrane expansion and maturation by forming a multi-layer compartment. Furthermore, double immunogold labeling of transgenic *SH3P2-GFP* plants using anti-SH3P2 and anti-ATG8a antibodies determined that the SH3P2-GFP–labeled multi-layer structures were autophagosomes or related structures (Zhuang et al. 2013). Through immunogold-EM studies, FYVE DOMAIN PROTEIN REQUIRED FOR ENDOSOMAL SORTING 1 (FREE1), a component of plant-specific endosomal sorting complex required for transport (ESCRT), was demonstrated to play an essential role in autophagic degradation (Gao et al. 2015), and ATG9 was shown to be essential for ER-derived autophagosome formation in plant cells (Zhuang et al. 2017).

While conventional TEM allows two-dimensional mapping of the labeled structures, three-dimensional tomographic reconstructions have proven to be useful for analyzing the complex membrane structures that participate in autophagy. Indeed, three-dimensional electron tomography analysis showed that phagophore membranes are connected with rough ER cisternae located inside nascent autophagosomes (Ylä-Anttila et al. 2009). In agreement, a recent study using electron tomography showed that the loss of ATG9 function led to a drastic accumulation of autophagosome-related tubular structures in direct membrane continuity with the ER upon autophagic induction in Arabidopsis (Zhuang et al. 2017). Ultrastructural TEM and three-dimensional electron tomography analyses showed that FREE1 is involved in autophagosome closure in Arabidopsis (Zeng et al. 2023). Furthermore, three-dimensional tomographic reconstruction showed that loss-of-function of UFM1-SPECIFIC E3 LIGASE 1 (Ufl1), an E3 ligase of the ufmylation system in Arabidopsis, leads to abnormal ER-phagy under salt stress conditions, indicating a role for Ufl1 in regulating ER homeostasis (Li et al. 2023).

More recently, another study employed three-dimensional tomographic reconstruction to elegantly demonstrate a noncanonical role for ATG8 in Golgi recovery from heat stress in plants (Zhou et al. 2023). Through immunogold-TEM, Arabidopsis OXYSTEROL-BINDING PROTEIN–RELATED PROTEIN 2A (ORP2A) was shown to localize alongside an autophagosome-like structure surrounded by the ER (Ye et al. 2022). ORP2A is involved in mediating ER–autophagosomal membrane contacts and autophagosome biogenesis according to three-dimensional electron tomography analysis and three-dimensional model reconstruction (Ye et al. 2022).

Therefore, TEM is an extremely powerful and accurate method for monitoring autophagy and represents the only technique with which to examine autophagy at subcellular resolution in diverse complex environments. However, TEM requires specialized equipment and expertise, making it challenging for many laboratories, and it cannot be used for imaging live cells. The use of TEM in combination with other technologies is becoming increasingly necessary to study the progression of autophagy.

#### Staining with fluorescent dyes

Although GFP-ATG8 is considered to be an ideal marker for autophagic structures, the use of the GFP-ATG8 fusion protein requires the transient expression of its encoding constructs in transfected protoplasts or its stable expression in transgenic lines. However, some acidotropic fluorescent dyes, such as autofluorescent amine monodansylcadaverine (MDC), Lysotracker Red (LTR), quinacrine, and Neutral Red, can be used to stain autophagosomes or autophagic bodies, making them fast and easy markers to stain wild-type and mutant plants for preliminary analysis of autophagosome formation (Moriyasu and Ohsumi 1996; Munafó and Colombo 2001; Yano et al. 2004; Contento et al. 2005; Liu et al. 2005; Inoue et al. 2006; Patel and Dinesh-Kumar 2008). Because mature autophagosomes have an acidic lumen, they accumulate acidotropic dyes.

MDC accumulates inside membrane compartments, such as autophagosomes, that have both an acidic lumen and a lipid-rich membrane, and fluorescence can be detected by confocal or standard fluorescence microscopy (Munafó and Colombo 2001; Contento et al. 2005). Under normal growth conditions, very few MDC-stained structures are observed in Arabidopsis suspension cells. Furthermore, most of the MDC-labeled punctate structures are found in the cytosol and colocalize with the autophagy marker GFP-ATG8e (Contento et al. 2005). Thus, MDC is considered to be an autophagosome-enriched marker in Arabidopsis, as shown in mammalian cells, and it has been extensively deployed to study autophagy in plants (Liu et al. 2005, 2009; Xiong et al. 2005; Patel and Dinesh-Kumar 2008; Chen et al. 2015; Huang et al. 2019a; Qi et al. 2017). Compared to other methods for analyzing plant autophagy, MDC offers a quick mean to stain Arabidopsis cells and entire seedlings without the need for stable transgenic plants.

LTR is another acidotropic fluorescent dye commonly used for detecting autophagosomes or autolysosomes in animals (Rodriguez-Enriquez et al. 2006). Recently, it was also used in the observation of autolysosomes in tobacco leaf cells treated with the cysteine protease inhibitor E-64d (Liu et al. 2005; Kwon et al. 2013). This dye stains the central vacuole weakly but stains smaller acidic compartments more strongly, and has been used in combination with cysteine protease inhibitors, such as E-64d, to allow accumulation of autophagosomes for easier visualization (Liu et al. 2005; Bassham 2015).

However, a recent study showed that MDC or LTR staining is not suitable for monitoring autophagy under some conditions in plants (Merkulova et al. 2014). In this study, very few MDC or LTR-stained structures were observed in the elongation zone of Arabidopsis roots under starvation conditions, in contrast to the abundant autophagosomes observed in *GFP-ATG8* transgenic lines grown under the same conditions. Moreover, there was no colocalization of MDC or LTR-positive vesicles with GFP-ATG8 fluorescence in root tips upon starvation (Merkulova et al. 2014). It is worth noting that this may be due to the shorter starvation treatment time in this study (Merkulova et al. 2014) compared to the longer starvation times traditionally used when studying autophagy in plants (Yoshimoto et al. 2004; Xiong et al. 2005; Phillips et al. 2008; Suttangkakul et al. 2011; Li et al. 2014; Bassham 2015; Huang et al. 2019b; Qi et al. 2017).

In summary, fluorescent dyes such as MDC and LTR should be used with caution, as they tend to stain other acidic compartments besides autophagosomes, and are best used for preliminary studies followed by alternative approaches such as GFP-ATG8 expression. Additional assays are required to assess MDC or LTR staining results before drawing conclusions about autophagy.

### Biochemical methods

Biochemical approaches offer an alternative means of assessing autophagic activity in eukaryotes. ATG proteins are widely used to monitor autophagic activity in plants; these include ATG1a, ATG13a, and ATG8, with ATG8 being the most widely used (Chung et al. 2010; Suttangkakul et al. 2011; Chen et al. 2015; Qi et al. 2017; 2020). In this section, we discuss multiple assays for monitoring autophagy using these ATG proteins.

#### Immunoblotting analysis of ATG proteins

##### ATG8 lipidation assay using anti-ATG8 antibodies

**Fig. 5:**
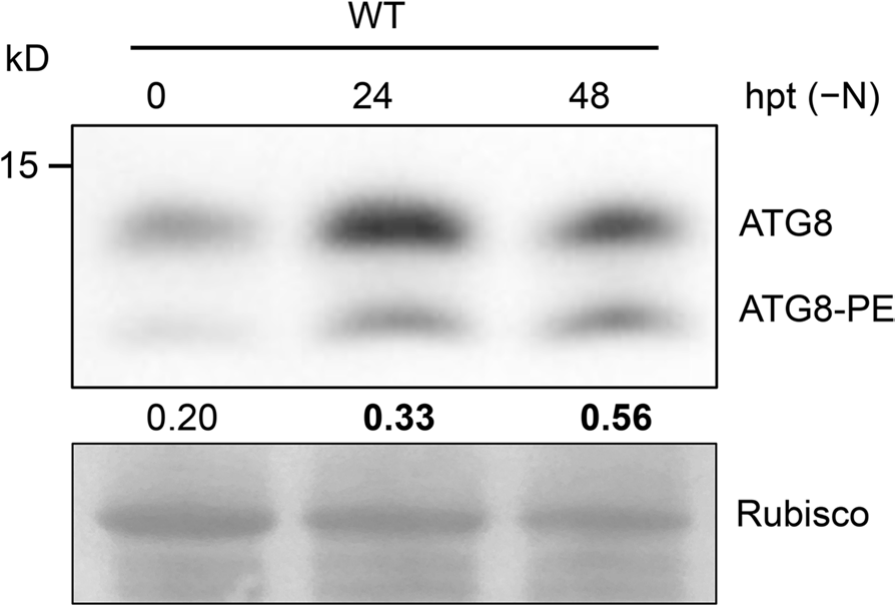
Analysis of autophagic activity via ATG8 lipidation. Total protein extracts were prepared from one-week-old wild-type (WT) seedlings exposed to nitrogen starvation (− N) for the indicated hours post-treatment (hpt). The total protein extracts were then subjected to SDS-PAGE in the presence of 6 M urea, followed by immunoblot analysis with antibodies against ATG8a. The ratio between ATG8–PE and ATG8 indicating the autophagy activity is shown below. Rubisco is shown below the blot to indicate the amount of protein loaded per lane

Autophagosome formation requires conjugation of ubiquitin-like ATG8 to PE (Ohsumi 2001; He and Klionsky 2009). ATG8 is post-translationally cleaved by the cysteine protease ATG4 at a conserved C-terminal glycine residue, thus leading to its conjugation with PE, forming ATG8–PE that localizes to the newly formed inner and outer membranes of autophagosomes (Ichimura et al. 2000; Chung et al. 2010; Woo et al. 2014). Although ATG8–PE on the outer membrane is recycled before the autophagosome fuses with the tonoplast, ATG8–PE on the inner membrane enters the vacuole together with the cargo for degradation (Mizushima et al. 2010). Indeed, the amount of ATG8–PE usually correlates with the number of ATG8-positive punctate structures as well as autophagic activity. Thus, the ATG8–PE adduct has been widely used as a biochemical marker for ATG-mediated autophagy in yeast, animals, and plants (Rubinsztein et al. 2009; Chung et al. 2010). The commercial availability of Arabidopsis anti-ATG8 antibodies has made it easy to determine autophagy activity in plants by comparing the ratio of lipidated to non-lipidated forms of ATG8 in Arabidopsis subcellular fractions (Chung et al. 2010; Gao et al. 2015; Fig. 5). Although the PE-conjugated form of ATG8 is larger in mass than free ATG8, it exhibits a faster electrophoretic mobility in SDS-PAGE gels, probably as a consequence of increased hydrophobicity (Klionsky et al. 2016).

Notably, the gels must be run in the presence of urea to analyze the lipidated and non-lipidated forms of ATG8 by immunoblotting (Klionsky et al. 2016). Furthermore, unlike the single-copy *ATG8* gene in yeast, the genomes of Arabidopsis and other plants contain multiple *ATG8* genes, resulting in several proteins similar in sequence and molecular mass (Chung et al. 2010). Thus, antibodies against ATG8a recognize most ATG8 isoforms, which differ substantially in terms of their mobility on SDS-PAGE, and their tendency to recognize unknown cross-reacting species that possess a fast mobility similar to that of ATG8–PE (Chung et al. 2010).

An effective solution to this problem is to introduce appropriate controls to distinguish between ATG8 and ATG8–PE. In the Arabidopsis *atg5* mutant, the level of ATG8–PE is severely decreased, with scarcely any ATG8-labeled autophagosome structures, whereas non-lipidated ATG8 accumulates in large amounts (Chung et al. 2010). Thus, it is critical to include wild-type and *atg5* mutant seedlings as positive and negative controls, respectively, to unequivocally identify ATG8–PE by immunoblotting analysis. Under conditions that induce autophagy, ATG8–PE adducts were absent in *atg7-2* membranes; however, they were abundant in wild type and several other autophagy-defective mutants, such as *atg9*, *atg13a atg13b* double mutants, and *atg11*, which show suppression of autophagic body deposition into the vacuole (Suttangkakul et al. 2011; Li et al. 2014; Zhuang et al. 2017).

To better identify lipidated ATG8, an improved method called the ATG8 delipidation assay was devised based on ATG8 membrane association and sensitivity to phospholipase D (PLD) in Arabidopsis (Chung et al. 2010). Immunoblotting analysis showed that the faster mobility species that were enriched in the membrane fractions from N-starved seedlings are sensitive to incubation with PLD, indicating that the faster mobility species contained phospholipids such as PE. However, the lipidated species detected in the membrane fraction of wild-type seedlings were absent in the *atg5*, *atg12a atg12b*, and *atg10* mutants (Chung et al. 2010). Furthermore, lipidated ATG8 species are converted to the non-lipidated form of ATG8 upon PLD digestion (Chung et al. 2010). Thus, it is easy to distinguish between ATG8–PE adducts and ATG8 by incubating protein extracts with PLD prior to immunoblotting alongside samples from wild-type and autophagy-defective mutant seedlings.

##### Monitoring autophagy by detecting ATG1a and ATG13a proteins

The ATG1 − ATG13 complex, one of the most upstream components of the autophagy machinery, plays an essential role in initiating autophagy by responding to nutritional status and governing autophagosome formation (Suttangkakul et al. 2011; Li et al. 2014). Notably, the ATG1 − ATG13 complex is both a regulator and target of autophagy, as it is degraded in an autophagy-dependent manner (Suttangkakul et al. 2011). This feature has made ATG1 − ATG13 a useful marker for detecting autophagy in plants. Previous studies have shown that anti-ATG1a and anti-ATG13a antibodies detect accumulation of both ATG1a and ATG13a in autophagy-defective mutants, such as *atg7*, *atg11*, and the *traf1a traf1b* double mutant (Li et al. 2014; Qi et al. 2017).

In plants, the stability or activity of the ATG1 − ATG13 complex is tightly controlled by posttranslational modifications, such as phosphorylation and ubiquitination (Qi et al. 2021; Wang and Hou 2022). For example, the SINAT family of RING-type E3 ubiquitin ligases differentially regulates ubiquitination and stability of the ATG1 − ATG13 complex to regulate autophagy (Qi et al. 2020; 2022a; 2022b). The plant energy sensors SnRK1 and TOR act as positive and negative regulators, respectively, of plant autophagy by modulating the phosphorylation of the ATG1 − ATG13 complex. The α-subunit of SnRK1 kinase, SNF1 KINASE HOMOLOG 10 (KIN10), mediates the phosphorylation of ATG1a in plants upon nutrient starvation to activate autophagosome formation (Chen et al. 2017a). Moreover, the TOR signaling (TOS) motif of ATG13 is important for its phosphorylation by TOR in Arabidopsis, indicating a potential role for TOR in the phosphorylation of ATG13 (Son et al. 2018). Furthermore, KIN10 functions upstream of TOR to activate autophagy, suggesting crosstalk between these two phosphorylation-based regulators of plant autophagy (Soto-Burgos and Bassham 2017). Recently, TYPE ONE PROTEIN PHOSPHATASE (TOPP) was shown to mediate the dephosphorylation of ATG13a during nutrient deprivation and increase tolerance to fixed-carbon starvation in Arabidopsis (Wang et al. 2022a, b). These results indicated that the phosphorylation status of the ATG1 − ATG13 complex is critical for autophagy in plants.

On immunoblots probed with anti-ATG1a antibodies, ATG1a was detected as a 70-kDa species close in size to the 69-kDa predicted molecular mass in wild-type plants, and this species accumulated in autophagy-defective mutants. At the same time, a protein that cross-reacted with anti-ATG1a antibodies was also detected with a molecular mass larger than 70 kDa. When incubated with λ phosphatase, the electrophoretic pattern of ATG1a appeared to shift from the lower mobility 72-kDa form to the higher mobility 70-kDa form, indicating that the 72-kDa and 70-kDa proteins represent the phosphorylated and non-phosphorylated forms of ATG1a, respectively (Suttangkakul et al. 2011).

When immunoblots were probed with anti-ATG13a antibodies, a diffuse ladder of three and sometimes four species that ranged in apparent molecular mass from 70 to 80 kDa was observed (Suttangkakul et al. 2011). The different species of ATG13a were produced not by alternative splicing of its mRNA, but rather by phosphorylation of a single 66-kDa translation product. Another band that cross-reacted with anti-ATG13a antibodies was detected with a molecular mass larger than 80 kDa. Treatment with λ phosphatase substantially reduced the levels of the 80-, 74-, and 70-kDa species of ATG13a, with the appearance of a new species at 66 kDa. Thus, the 66-kDa species is likely the non-phosphorylated form of ATG13a, whereas the 70–80-kDa species represent ATG13a with different levels of phosphorylation (Suttangkakul et al. 2011).

#### Analyzing plant autophagy by detecting NBR1 protein

Autophagy was initially considered to be a non-specific process that is induced by a number of biotic and abiotic stresses (Bassham 2015). However, increasing evidence showed that autophagy also regulates cellular homeostasis by selectively degrading specific cargos (Stolz et al. 2014). The selective autophagy receptors recognize their target proteins specifically and recruit them into double membrane–bound autophagosomes for breakdown by associating with ATG8 proteins (Stolz et al. 2014). Specific interactions between selective autophagy receptors and ATG8 require the conserved ATG8-interacting motif (AIM) and ubiquitin-interacting motifs (UIM) within the selective autophagy receptors (Johansen and Lamark 2011; Marshall et al. 2019).

A selective autophagy receptor has been identified in plants, termed NEIGHBOR OF BRCA 1 (NBR1) in Arabidopsis or Joka2 in tobacco; this protein is a structural homolog and functional hybrid of the mammalian autophagy receptors NBR1 and p62 (Svenning et al. 2011; Zientara-Rytter et al. 2011). During selective autophagy, NBR1 enters the vacuole together with its substrate proteins for degradation and accumulates in autophagy loss-of-function mutants (Svenning et al. 2011; Jung et al. 2020; Thirumalaikumar et al. 2021). To monitor the autophagic sequestration of NBR1 in Arabidopsis, the fluorescence localization differentiation of NBR1 fusions with acid-insensitive mCherry or the acid-sensitive YFP was observed by confocal microscopy, revealing that NBR1 is an autophagy substrate degraded in the vacuole (Svenning et al. 2011). Consistent with this observation, increased NBR1 accumulation was found in the Arabidopsis *atg7* mutant in comparison with wild-type plants (Svenning et al. 2011). Thus, NBR1 itself is a substrate for autophagy recycled along with its cargo during the process of selective clearance, which also reflects the autophagic flux to some extent in plants.

#### Cleavage of GFP-ATG8e for analyzing plant autophagy

**Fig. 6:**
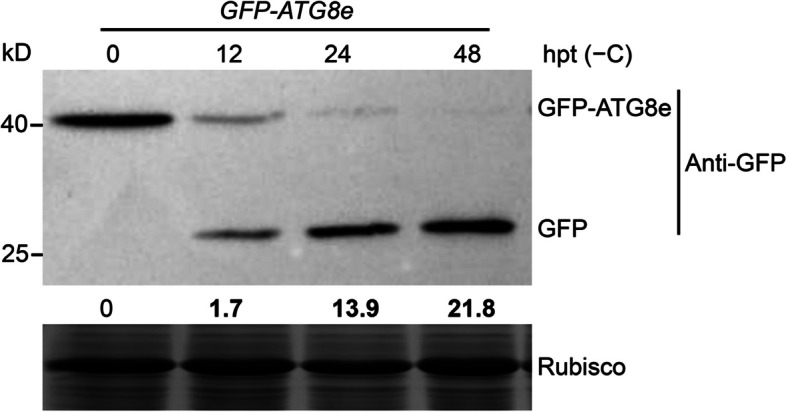
Immunoblot analysis showing processing of the GFP-ATG8e fusion upon carbon starvation. One-week-old *GFP-ATG8e* seedlings were transferred to MS medium without sucrose (− C) for the indicated times before protein extraction. Crude extracts were subjected to SDS-PAGE and immunoblot analysis with anti-GFP antibodies. GFP-ATG8e and free GFP are indicated on the right. The ratio between free GFP and GFP-ATG8e is shown below. Coomassie blue-stained total proteins are shown below the blots as a loading control. hpt, hours post treatment

Upon induction of autophagy, ATG8 − PE conjugates decorate the inner and outer membranes of autophagosomes (Yoshimoto et al. 2004; Woo et al. 2014). ATG8–PE on the outer membrane is recycled before the autophagosome fuses with the tonoplast, while the ATG8–PE at the inner membrane will enter the vacuole (Mizushima et al. 2010). Although GFP is acid-sensitive and is rapidly degraded in lysosomes (pH 4.5), it is degraded more slowly in the plant vacuole with its slightly higher pH (pH 5.4–5.8) (Kaizuka et al. 2016; Liu et al. 2022). For this reason, GFP-ATG8 is commonly used not only for visualizing autophagosomes by confocal microscopy but also for biochemical methods using anti-GFP antibodies. Upon induction of autophagy, free GFP accumulates in a time-dependent manner in the vacuole as GFP-ATG8 disappears (Fig. 6), and both of these changes are blocked in autophagy-defective mutants. The ratio of free GFP to GFP-ATG8 thus reflects autophagic flux (Chen et al. 2015; Qi et al. 2017; Huang et al. 2019b).

### Identification of key regulators of plant autophagy

Many key components of the core autophagy machinery have been identified in plants. Nevertheless, identification of new players in plant autophagy is extremely important for further understanding the autophagy signaling network. Multi-omics approaches, such as genetic and functional analyses of the interactome of ATG proteins, genome-wide transcriptome profiling, and proteomics approaches, may help reveal additional key components of autophagy and increase our understanding of the associated regulatory network.

#### Interactome of ATG proteins for analyzing plant autophagy

ATG8 not only is a critical component for the formation of autophagosomes in plants but also is involved in specific association with multiple protein targets to regulate their selective turnover by the autophagy machinery (Marshall and Vierstra 2018). Even though these cargo proteins otherwise exhibit low sequence similarity to each other, they contain a common AIM, generally referred to as a W/YXXL/I/V-like motif, for direct binding with ATG8 family proteins (Liu et al. 2021). Because of this property, ATG8 has been a key molecule in proteomics-based studies of autophagy.

Using the Arabidopsis ATG8f isoform as the bait in a yeast two-hybrid (Y2H) approach, several positive cDNA clones were identified, including two proteins with AIM domains, named ATG8-INTERACTING PROTEIN 1 (ATI1) and ATI2 (Honig et al. 2012). These two proteins are involved in seed germination in response to exogenous abscisic acid (ABA) treatment, indicating a potential link between autophagy and ABA signaling.

A recent study used GFP-trap-based pull-down assays and large-scale proteomics analyses to look for proteins that interacted with YFP-ATG6 and YFP-ATG8 in transgenic Arabidopsis plants upon nutrient deprivation. This study identified two core components of the coat protein complex II (COPII) machinery, indicating a possible connection between the ATG machinery and specific COPII components in plants (Zeng et al. 2021). Further analysis showed that the COPII components Sec23b and Sec23f are associated with ATG6, while another component of COPII, Sar1d, specifically recognizes ATG8e via a noncanonical motif to regulate autophagosome progression (Zeng et al. 2021). Another GFP-trap-based pull-down assay using rice (*Oryza sativa*) *GFP-ATG8a* transgenic lines also identified several ATG8a-interacting proteins upon NaCl treatment, providing potential connections between autophagy and salt stress tolerance in rice (Liu et al. 2022).

A Y2H study using *Nicotiana tabacum* ATG6 as bait identified additional regulators of autophagy. BAX INHIBITOR-1 (BI-1), a highly conserved cell death regulator, interacts with ATG6 to regulate plant autophagy and programmed cell death (Xu et al. 2017). Β-TUBULIN 8, the major components of microtubules, was identified as an ATG6 interactor and further analysis showed that ATG6 colocalizes with microtubules. Disruption of microtubules suppresses autophagy (Wang et al. 2015). These results indicate that an intact microtubule network is important for efficient autophagy and leaf starch degradation.

Furthermore, mass spectrometry (MS)-based assays identified glyceraldehyde-3-phosphate dehydrogenases (GAPCs) as ATG3-interacting proteins; GAPCs regulate autophagy and programmed cell death during innate immune responses in *Nicotiana benthamiana* (Han et al. 2015). Thus, MS-based proteomics approaches have proven to be a powerful tool for confirming the assembly of higher-order complexes and the binding of ATG8 to autophagic cargo receptors.

#### Genome-wide transcriptome profiling for revealing the function and regulatory mechanism of plant autophagy

In plants, alteration of *ATG* gene expression helps plants to proceed through various specific developmental stages and respond to various environmental cues, thus promoting plant growth or survival (Yang et al. 2020a). Studies using DNA microarrays showed that several *ATG* genes were transcriptionally activated in naturally senescing or dark-incubated leaves in Arabidopsis (Buchanan-Wollaston et al. 2005; van der Graaff et al. 2006; Breeze et al. 2011), indicating that activation of autophagy plays a role in nutrient re-mobilization during leaf senescence. Microarray assays using the Arabidopsis wild-type and *atg* mutants revealed that genes involved in salicylic acid and ethylene biosynthesis were up-regulated in *atg* mutants compared to wild-type plants, which is consistent with the increased levels of these phytohormones and the early senescence phenotype found in *atg* mutants (Masclaux-Daubresse et al. 2014). Transcriptome analysis also showed that the NAC transcription factor TRANSCRIPTION ACTIVATION FACTOR (ATAF1) is involved in regulating the expression of *ATG* genes during carbon starvation–induced senescence in Arabidopsis. Genetic evidence showed that loss of ATAF1 results in decreased autophagic activity in Arabidopsis, suggesting that ATAF1 may act as a key regulator that integrates energy supply with *ATG* gene expression (Garapati et al. 2015). These results imply that as a key pathway for nutrient recycling, many plant *ATG* genes are upregulated during nutrient deprivation conditions, as well as during leaf senescence, and are most likely regulated by upstream transcription factors.

Genome-wide transcriptome studies also can help researchers investigate the function of autophagy in plant growth and development. RNA-seq analysis revealed that several *ATG* genes were up-regulated in the endosperm during seed development in maize (Li et al. 2015). A more recent study confirmed that *ATG* gene expression is strongly induced during silique development in Arabidopsis, and that *atg* mutant plants showed increased rates of seed abortion and altered deposition of seed storage proteins in the viable seeds (Di et al. 2018). Although numerous studies showed that *ATG* genes are transcriptionally regulated during plant growth and development, the underlying molecular mechanisms remain to be elucidated.

Increasing evidence demonstrates that the expression of *ATG* genes is upregulated to activate autophagy and maintain cellular homeostasis under a wide range of stress conditions (Yang et al. 2020a, b). Global transcriptome analysis found that transcripts of *ATG* genes were more abundant during periods of desiccation in plants (Williams et al. 2015; Zhu et al. 2015). Additionally, transcriptomic analysis revealed an increase of *ATG* gene expression in *Chlamydomonas reinhardtii* (a unicellular green alga) treated with nickel (Pérez-Martín et al. 2015), indicating a role of autophagy in plant tolerance to heavy metals. To date, a number of transcription factors such as WRKY, NAC, HEAT SHOCK FACTOR A1a (HsfA1a), BRASSINAZOLE RESISTANT 1 (BZR1), ELONGATED HYPOCOTYL (HY5), MOTIF-BINDING PROTEIN 9 (TGA9), and ETHYLENE RESPONSE FACTOR 5 (ERF5), have been identified as involved in stress responses (Yang et al. 2020a, b). These transcription factors are induced or repressed to activate the gene expression of downstream *ATG* genes through binding the specific cis elements in their promoters, thereby stimulating autophagic activity to enhance plant acclimation to growth conditions (Yang et al. 2020a, b).

Transcriptional profiles also provide valuable insights into the regulatory mechanism of plant autophagy (Liu et al. 2018). In Arabidopsis, L‐CYS DESULFHYDRASE (DES) catalyzes the enzymatic desulfuration of L‐Cys to sulfide and *des1* mutants exhibit decreased H_2_S production in the cytosol and increased accumulation of lipidated ATG8–PE conjugates (Alvarez et al. 2012). Transcriptional profiles of *des1* mutants grown with or without exogenous sodium sulfide (Na_2_S) led to the conclusion that sulfide represses autophagy (Álvarez et al. 2012). A global transcriptome analysis in Arabidopsis also confirmed that TOR kinase functions as a negative regulator of plant autophagy (Caldana et al. 2013). Thus, genome-wide transcriptome profiling revealed the fundamental function and regulatory mechanism of plant autophagy, although the underlying mechanisms will require further study.

#### Proteomics assay for analyzing plant autophagy

Thus far, mass spectrometry (MS)-based proteomics has proven to be a powerful tool for identification of changes in protein abundance under stress conditions to reveal the regulatory mechanisms of autophagy. Comparative proteomics assays analyzing protein contents of wild-type and *atg* mutant in Arabidopsis have found that autophagy is involved in protein degradation during plant development and responses to biotic and abiotic stresses (Avin-Wittenberg et al. 2015, Wang et al. 2018; Thirumalaikumar et al. 2021). Furthermore, increasing evidence demonstrates that the activities and stabilities of ATG proteins are strongly affected by regulatory posttranslational modifications such as phosphorylation, ubiquitination, lipidation, S‐sulfhydration, S‐nitrosylation, and acetylation, during autophagosome formation in plants (Qi et al. 2021). However, the application of proteomics in plant autophagy, like post-translational modification omics of ATGs proteins has yet to be reported. Such approaches have great potential for uncovering additional regulators of plant autophagy.

Therefore, it is important to build models of the regulatory networks, by which autophagy acts in plant development and responses to different stress stimuli, for us to understand how autophagy integrates multiple environmental cues in plant cells by coordinating multiple omics techniques.

## Recommendations for analyzing plant autophagy

### Plant materials and growth conditions

1. Arabidopsis seeds are surface-sterilized with 20% (v/v) bleach containing 0.1% (v/v) Tween-20 for 20 min and washed at least five times with sterile water.
2. The seeds are sown on solid Murashige and Skoog (MS) medium containing 1% (w/v) sucrose and 0.8% (w/v) agar, followed by stratification at 4°C in the dark for 3 days.
3. After incubation at 22°C under a long-day (LD,16-h light/8-h dark) photoperiod for 7 days, the seedlings are transferred to soil and grown under LD or short-day (SD, 8-h light/16-h dark) conditions for further growth and analysis. If there is no specific explanation, the plant growth conditions in the following protocols are the same as mentioned in this section.

NOTE: Reagents used in the protocols are shown in Supplemental Table 1.

### PROTOCOL 1. Phenotypic analysis of natural and starvation-induced senescence

#### Senescence phenotype analysis

1. Seven-day-old wild-type or autophagy-deficient mutant seedlings are transferred to soil and grown under LD or SD conditions for 6 (LD) or 8 (SD) weeks.
2. During this period, the onset and development of the senescence phenotype is recorded by photography and measurement of chlorophyll contents once a week.
3. Chlorophyll contents are measured as previously described (Xiao et al. 2010). Total chlorophylls are extracted from the rosettes of one plant with 5 technical replicates by immersion in 5 mL ethanol for 48 h in the dark at 4°C. Absorbance is determined at 664 nm and 647 nm, and the total chlorophyll concentration is calculated and normalized to grams of fresh weight per sample. The values for 3-week-old plants of different genotypes are set to 100% and the relative chlorophyll contents for these genotypes at other stages are normalized to this value.

#### Fixed-carbon starvation treatment of adult plants

1. For fixed-carbon starvation treatments of adult plants, 7-day-old seedlings are transferred to soil and grown under LD conditions for another two weeks (+C).
2. The resulting three-week-old plants are transferred to complete darkness for 8 d (−C) and allowed to recover under LD conditions for another 7 d (recovery).
3. Samples are collected or photographed at the appropriate time points.
4. The survival rates are calculated after a 7-d recovery following dark treatment, and the chlorophyll contents are measured at appropriate dark treatment time points. The number of surviving plants is recorded, as indicated by their ability to produce new leaves. The values for 3-week-old plants with different genotypes before dark treatment are set to 100% and the relative chlorophyll contents following different dark durations are normalized to these values.

#### Fixed-carbon starvation treatment on seedlings

1. For fixed-carbon starvation treatment of seedlings, 1-week-old MS-grown seedlings are transferred to MS agar plates with sucrose (+C) or MS agar plates without sucrose, followed by constant dark treatment (–C) for about 7 to 10 days until the sensitive seedlings start showing poor growth.
2. After recovery under normal growth conditions for 7 days, seedling phenotypes are recorded by photography.
3. The survival rate after fixed-carbon starvation is calculated from 15 seedlings per genotype and is defined as the percentage of seedlings with obvious regreening and the appearance of new leaves. The chlorophyll contents are measured and calculated as above. The relative chlorophyll contents are calculated by comparing the values of –C and +C seedlings.

#### Nitrogen starvation treatment

1. For N starvation treatment in liquid medium, 1-week-old seedlings grown on solid MS medium are transferred to 2 mL of liquid MS medium (+N) or N-free liquid MS medium (–N) in a 12-well tissue culture plate (30–60 seeds per well) for 4–5 days under LD conditions. Seedlings grown in liquid MS medium (+N) are used as the controls.
2. For N starvation treatment on solid medium, 1-week-old seedlings grown on solid MS medium are transferred to solid MS medium (+N) or N-free MS agar medium (–N) and incubated under LD conditions for 5–7 days.
3. Photographs are taken, and chlorophyll contents are measured when significant yellowing of leaves and anthocyanin accumulation are observed. The relative chlorophyll contents are calculated by comparing the values of –N and +N seedlings.

### PROTOCOL 2. Microscopy analysis of GFP-ATG8e labeled punctate structures

1. To detect autophagosomes or autophagic bodies using the GFP-ATG8e fusion protein, the *GFP-ATG8e* reporter is introduced into wild-type or other genotype lines by crossing them with transgenic plants harboring the *GFP-ATG8e* transgene, followed by selection of lines that are homozygous for both the mutation of interest and the transgene.
2. Seven-day-old *GFP-ATG8e* seedlings are transferred to liquid MS medium without sucrose, followed by incubation in continuous dark (−C) or N-deficient liquid MS medium (−N) with 1 μM ConA (optional) for 16−24 h.
3. After the seedlings are transferred to a glass slide with water, a coverslip is placed on top of the root. Importantly, the roots should lie straight along the slide, and bubbles in the water should be avoided when the coverslip is placed on top of the roots.
4. Cells within the elongation zone of the primary root are observed using a laser scanning confocal microscope with a 40 × objective lens. GFP is excited at a wavelength of 488 nm produced by an argon/krypton laser and detected with a bandpass 500−530 nm filter.
5. When viewed through the confocal microscope, the GFP-marked autophagosomes and autophagic bodies can be identified as punctate structures 1−2 μm in size in the cytoplasm and vacuole.
6. The number of GFP-ATG8e-labeled autophagosomes is counted in each frame and the average number of foci is calculated across all images for each genotype or treatment.

### PROTOCOL 3. Immuno-EM labeling for autophagosome observation

The general procedures for preparing samples for TEM have been previously described (Lin and Zhuang 2017).

1. Four- or five-day-old *YFP-ATG8e* transgenic seedlings grown under LD conditions are transferred to liquid MS medium containing 100 μM BTH and 1 μM ConA for at least 6 h.
2. High-pressure freezing: 5-day-old Arabidopsis transgenic root tips are cut into lengths of 3–4 mm from seedlings treated with 100 μM BTH and placed into high-pressure freezing (HPF) planchettes filled with 0.15 M sucrose solution. The planchette sandwich, along with the Arabidopsis root tips, is frozen immediately in an HPF apparatus (Leica, EM PACT2).
3. Freeze-substitution: For immunogold labeling, subsequent freeze-substitution is conducted in dry acetone containing 0.1% (w/v) uranyl acetate at −85°C for 48 h to replace frozen crystalline and noncrystalline water in the root tips. The temperature is gradually increased to –50°C over a 30-h time frame, followed by infiltration with Lowicryl HM20 resin step-by-step with the changes of resin in ethanol (0%, 33%, 66%, and 100%, all v/v) each for 1 h. Infiltration with HM20, embedding, and UV polymerization are performed stepwise at –35°C.
4. After polymerization, mounting, trimming, and ultramicrotomy are performed as described in comprehensive books about EM (Hagler 2007; Lin and Zhuang 2017).
5. Immunogold labeling: Fixed roots are incubated with anti-GFP antibody at 4 µg/mL overnight at 4 °C or incubated at room temperature for 1–4 h for immunolabeling before being probed with gold particle–coupled (various sizes: 6, 10, and 15 nm) secondary antibodies in solution (1: 40; 15–30 μL per sample) with incubation for 45–60 min at room temperature. Three 30-μL drops of washing solution for each sample are used and samples are washed three times for 5–10 min each. The samples are dried and stained with aqueous uranyl acetate/lead citrate, followed by observation of labeled autophagosome structures under TEM. As shown in Fig 4, the anti-GFP gold particles (15 nm) label double membrane-bound structures.

### PROTOCOL 4. MDC staining for monitoring autophagy in plants

1. Seven-day-old MS-grown seedlings are transferred to liquid MS medium without sucrose and are incubated under continuous dark conditions (–C) or N-free liquid MS medium (–N) for 16–24 h.
2. Seedlings are stained with 50 μM MDC in 1 × phosphate buffered saline (PBS) for 10 min.
3. After incubation, seedlings are washed four times with 1 × PBS.
4. The seedlings are transferred to a glass slide with water; a cover glass is placed on top of the seedling from the edge of the root to ensure no air bubbles on the slide. For convenience, the root is placed straight along the slide.
5. Autophagosomes are observed using a confocal microscope with a 40× oil-immersion objective. MDC fluorescence is excited at a wavelength of 335 nm and detected at 400–580 nm.
6. Autophagosomes can also be observed using a fluorescence microscope with a 4,6-diamino-phenylindole (DAPI)-specific filter.

### PROTOCOL 5. ATG8 lipidation and delipidation assays

#### Protein extraction

1. Seven-day-old MS-grown seedlings are transferred to MS medium without sucrose and incubated under continuous dark conditions (–C) or N-free MS liquid medium and incubated under normal light conditions (–N). Approximately 200 mg of seedlings is collected at the appropriate time.
2. The samples are ground thoroughly into powder with liquid nitrogen in 1.5-mL microcentrifuge tubes with a pestle.
3. To each ground sample, 200 μL ice-cold protein extraction buffer with protease inhibitor cocktail (Roche) is added; the samples are homogenized on ice for 30 min.
4. The samples are centrifuged at 1,000 ×*g* for 5 min at 4℃.
5. The supernatant is transferred to new tubes and centrifuged at 100,000 ×*g* for 60 min at 4℃.
6. The supernatant is transferred (Cell Soluble fraction, CS) to a new 1.5-mL microcentrifuge tube and kept on ice.
7. The pellet (Cell Membrane fraction, CM) is washed gently with protein extraction buffer containing protease inhibitor cocktail (Roche) 2–3 times to remove the remaining CS liquid on the surface of the CM pellet. The protein extraction buffer should be added gently from the side of the tube rather than poured directly onto the pellet
8. The pellet is resuspended in protein extraction buffer containing a protease inhibitor cocktail (Roche) and 1% (v/v) Triton X-100 to solubilize the membranes.
9. The resuspended membranes are divided among three tubes: one is kept on ice (control), one is incubated for 1 h with phospholipase D (250 units/mL), and one is incubated for 1 h with PLD buffer only.
10. 5 × SDS sample loading dye is added to the CS and CM samples, before boiling the samples at 95 °C for 10 min.

#### Preparation of the SDS-PAGE gel with urea

1. The gel plates are washed and dried. To increase the migration distance of different proteins, the vertical dimensions of the plates should be greater than 12 cm.
2. 3.6 g urea (molecular biology grade) is weighed, and added to a 50-mL centrifuge tube.
3. 3.8 mL of 1 M Tris base (pH 8.8) and 5 mL of 30% (w/v) acrylamide solution are added and the urea is dissolved by vortexing. Urea should be dissolved before the SDS is added owing to the production of bubbles when vortexing solutions containing SDS.
4. 0.1 mL of 10% (w/v) SDS, 0.1 mL of 10% (w/v) ammonium persulfate (APS), and 4 μL of *N*,*N*,*Nʹ*,*Nʹ*-tetramethylethylenediamine (TEMED), and the appropriate amount H_2_O are added to make the final volume to 10 mL.
5. The mixture is mixed and poured between the gel plates.
6. After polymerization of the resolving gel, a standard stacking gel is prepared without urea.

#### Electrophoresis and immunoblotting

1. 30 μL of the protein samples are loaded into each well of a urea gel, which is run at room temperature and 90 V for 2 h or stopped when the dye front reaches the bottom of the gel.
2. The proteins from the gel are transferred to Hybond-C membrane (Amersham) with 0.22-μm pore diameter at 200 mA for 1 h at 4 °C in 1 × transfer buffer.
3. After transfer, the membrane is cut and the region containing proteins from 10 to 20 kDa is used for immunoblotting against ATG8. The other part of the membrane, specifically that near Rubisco at around 55 kDa, can be used for Ponceau S staining to provide a loading control.
4. The membrane is blocked in 1× Tris-buffered saline (TBS) with 0.1% Tween-20 (TBST) containing 5% (w/v) nonfat milk powder for 1 h with gentle shaking. And then, the membrane is washed with 1× TBST three times, once for 10 min.
5. The anti-ATG8 antibody (cat. No. ab77003, Abcam) is diluted in 1× TBST buffer (1:1,000) and incubated with the membrane for 2 h at room temperature. The membrane is then washed with TBST 3 times.
6. The membrane is incubated with the secondary antibody (HRP-conjugated Affinipure Goat Anti-Rabbit IgG (H+L); Cat No: SA00001-2, proteintech;1: 5,000 in 1× TBST buffer) for 1 h and washed with TBST 3 times.
7. Western blotting detection reagents are added to the membrane, and ATG8 and ATG8 − PE bands are detected by chemiluminescence.

### PROTOCOL 6. GFP-ATG8 cleavage assay

1. Seven-day-old MS-grown *GFP-ATG8e* transgenic seedlings are transferred to sucrose-free solid MS medium and incubated under continuous dark conditions (–C) or N-free MS medium under normal light conditions (–N) for 0, 6, 12, or 24 h. Approximately 200 mg of seedlings is collected at appropriate time points during treatment.
2. The samples are ground thoroughly into powder with liquid nitrogen and homogenized in ice-cold extraction buffer containing protease inhibitor cocktail (Roche) in 1.5-mL microcentrifuge tubes.
3. The samples are incubated on ice for 30 min and centrifuged at 4°C for 30 min at 12,000g. The supernatant is transferred to a new microfuge tube containing 5×SDS sample loading dye for denaturation at 95°C, prior to electrophoresis.
4. A 12% (w/v) resolving gel and 5% (w/v) stacking gel is prepared.
5. 30 μL of each protein sample as well as protein markers are loaded onto the gel, which is then run at a constant 90 V for 2–3 h at room temperature until the dye front reaches the bottom of the gel.
6. After electrophoresis, the resolving gel is cut from 20 to 55 kDa for transfer to membrane, as described above, followed by blocking in 5% (w/v) nonfat milk dissolved in 1× TBST for at least 1 h. The membrane is washed with TBST three times.
7. The membrane is incubated with anti-GFP primary antibody (cat. no. M20004, Abmart, Shanghai, China, diluted 1:3000 in 1×TBST) for 2 h and washed with TBST 3 times for 10 min each time.
8. The membrane is incubated with the secondary antibody (diluted 1:5,000 in 1×TBST) for 1 h and washed with TBST buffer 3 times for 10 min each time.
9. The GFP-ATG8e fusion protein and free GFP bands are detected by chemiluminescence as described above.
10. Quantification of the protein signal is done using Image J software, and the relative intensity of each protein is normalized to the loading control.

## Conclusions

In this review, we have summarized and evaluated frequently used assays for assessing autophagy activity in plant cells. Assays based on fluorescence microscopy are widely used and convenient because the GFP-ATG8 fusion is an excellent marker for monitoring autophagy in plants and can be used in stable transgenic plants and transiently transfected protoplasts. Owing to its high resolution, TEM is an extremely powerful method for detecting the ultrastructure of autophagic structures and identifying cargo during autophagy. Combined with other techniques, such as immunogold labeling and three-dimensional tomographic reconstruction, TEM has been used to study the morphology of autophagosomes and autophagic bodies and to explore key regulators of autophagy in plants. Biochemical techniques analyzing the accumulation of ATG proteins, especially ATG8, have become the main methods for measuring autophagic activity owing to the commercial availability of anti-ATG antibodies. In addition to Arabidopsis, researchers have used rice, *Nicotiana benthamiana*, and *Chlamydomonas* as experimental models to study autophagy (Klionsky et al. 2016). *Chlamydomonas* has unique advantages; for example, its ATG machinery is encoded by single-copy genes (Pérez-Pérez et al. 2010; Klionsky et al. 2016). However, *Arabidopsis thaliana* remains a favored model because of its well-characterized genetics, and the feasibility of transient expression of any gene of interest (Marion et al. 2018). It should be noted that, most of these techniques have been used in either Arabidopsis or *N. benthamiana* cell cultures; whether they will be effective in other plant species needs to be established. Furthermore, multiple assays should be combined when analyzing autophagy activity, as no single autophagy assay is fully conclusive under all conditions. The development of more powerful methods for autophagy analysis will likely lead to new insights into the mechanism, regulation, and physiological effects of autophagy over the next few years.

## Plant ethics

The mutants we used in this study are from the ABRC (www.arabidopsis.org), and all analyses were performed under laboratory conditions.

## Supplementary Information

**Additional file 1:** **Table 1. **Reagents used in protocols.

## Data Availability

The authors declare that all data supporting the findings of this study can be found within the paper. Additional data supporting the findings of this study are available from the corresponding author upon request.
