# Supplementary material for: Studying plant autophagy: challenges and recommended methodologies
Source: Adv Biotechnol (Singap). 2023 Oct 26;1(4):2. doi: 10.1007/s44307-023-00002-8 (PMC11727600; doi:10.1007/s44307-023-00002-8)
Supplement: Supplementary file 1 — Additional file 1: Table 1. Reagents used in protocols. [file 44307_2023_2_MOESM1_ESM.docx]

**Supplemental Table 1**. **Reagents used in protocols**

| **Regents in Protocol 1** | |
| --- | --- |
| Reagent 1 | Solid MS medium: 4.4 g/L Murashige and Skoog Basal Salt Mixture (Sigma-Aldrich, cat. no. M5519), 20 g/L sucrose (Sigma-Aldrich, cat. no. S9378), pH 5.8 with NaOH, 0.8% agar (Sigma-Aldrich, cat. no. A1296) to prepare solid MS medium. |
| Reagent 2 | Liquid MS medium: 4.4 g/L Murashige and Skoog Basal Salt Mixture (Sigma-Aldrich, cat. no. M5519), 20 g/L sucrose (Sigma-Aldrich, cat. no. S9378), pH 5.8 with NaOH, 0.8% to prepare liquid MS medium. |
| Reagent 3 | Solid MS medium without sucrose: 4.4 g/L Murashige and Skoog Basal Salt Mixture (Sigma-Aldrich, cat. no. M5519), pH 5.8 with NaOH, 0.8% agar (Sigma-Aldrich, cat. no. A1296) to prepare solid MS–C medium. |
| Reagent 4 | Solid MS medium without nitrogen: 4.4 g/L Murashige and Skoog Basal Salt Mixture without nitrogen (Caisson, cat. no. MSP07), 20 g/L sucrose (Sigma-Aldrich, cat. no. S9378), pH 5.8 with NaOH, 0.8% agar (Sigma-Aldrich, cat. no. A1296) to prepare solid MS–N medium. |
| Reagent 5 | Liquid MS medium without sucrose: 4.4 g/L Murashige and Skoog Basal Salt Mixture (Sigma-Aldrich, cat. no. M5519), pH 5.8 with NaOH to prepare solid MS–C medium. |
| Reagent 6 | Liquid MS medium without nitrogen: 4.4 g/L Murashige and Skoog Basal Salt Mixture without nitrogen (Caisson, cat. no. MSP07), 20 g/L sucrose (Sigma-Aldrich, cat. no. S9378), pH 5.8 with NaOH to prepare liquid MS–N medium. |
| Reagent 7 | Soil mixture: Peat moss and vermiculite (4:1 mix) presoak with distilled water and then autoclave sterilized for 30 min. |
| **Regents in Protocol 2** | |
| Reagent 1 | Solid MS medium: See Reagent 1 in protocol 1. |
| Reagent 2 | Liquid MS medium: See Reagent 2 in protocol 1. |
| Reagent 3 | Liquid MS–C medium: See Reagent 5 in protocol 1. |
| Reagent 4 | Liquid MS–N medium: See Reagent 6 in protocol 1. |
| Reagent 5 | ConA stock: 1 mM stock solution is prepared by dissolving ConA (APExBIO, cat. no. A8633) with DMSO (Sigma-Aldrich, cat. no. D5879). |
| **Regents in Protocol 3** | |
| Reagent 1 | Solid MS medium: See Reagent 1 in protocol 1. |
| Reagent 2 | Liquid MS medium: See Reagent 2 in protocol 1. |
| Reagent 3 | BTH stock: 10 mM stock solution is prepared by dissolving BTH (Supelco, cat. no. 32820) with methanol. |
| Reagent 4 | ConA stock: See Reagent 5 in protocol 2. |
| Reagent 5 | Sucrose solution: 0.15 M sucrose solution. |
| Reagent 6 | Freeze substitution solution: 0.1% uranyl acetate in dry acetone. |
| Reagent 7 | 10 × phosphatebuffered saline (PBS) stock: 80 g/L NaCl, 2 g/L KCl, 11.4 g/L Na_2_HPO_4_·H2O, and 2 g/L KH_2_PO_4_, pH 7.3. |
| Reagent 8 | Blocking solution: 3% BSA in 1 × PBS solution (dissolved from 10 × PBS solution), which is filter-sterilized before used. |
| Reagent 9 | Washing solution: 1% BSA in 1× PBS solution (dissolved from 10 × PBS solution), which is filter-sterilized before used. |
| Reagent 10 | GFP antibodies: Dilute the anti-GFP antibodies (Abmart, cat. no. M20004) with filter-sterilized 1% BSA to 40 μg/ml. |
| Reagent 11 | Secondary antibody: Gold particle-coupled (various sizes: 6, 10, and 15 nm) secondary antibodies against the host of the primary antibodies are diluted to 1: 40 before use. |
| **Regents in Protocol 4** | |
| Reagent 1 | Solid MS medium: See Reagent 1 in protocol 1. |
| Reagent 2 | Liquid MS medium: See Reagent 2 in protocol 1. |
| Reagent 3 | Liquid MS–C medium: See Reagent 5 in protocol 1. |
| Reagent 4 | Liquid MS–N medium: See Reagent 6 in protocol 1. |
| Reagent 5 | MDC stock: MDC (Sigma-Aldrich, cat. no. 30432) is dissolved by [methanol](javascript:;) to 1 mM. |
| Reagent 6 | ConA stock: See Reagent 5 in protocol 2. |
| Reagent 7 | 1 × PBS: Prepared from 10 × PBS (See Reagent 7 in protocol 4) by dilution. |
| **Regents in Protocol 5** | |
| Reagent 1 | Solid MS medium: See Reagent 1 in protocol 1. |
| Reagent 2 | Liquid MS medium: See Reagent 2 in protocol 1. |
| Reagent 3 | Liquid MS–C medium: See Reagent 5 in protocol 1. |
| Reagent 4 | Liquid MS–N medium: See Reagent 6 in protocol 1. |
| Reagent 5 | Protein extraction buffer: 250 mM Tris-HCl pH 7.4, 750 mM NaCl, 5 mM EDTA supplemented with protease inhibitor cocktail (Roche, cat. no. 04693132001). |
| Reagent 6 | Triton X-100: Sigma-Aldrich, cat. no. T8787. |
| Reagent 7 | Phospholipase D: Prepared from [*Streptomyces chromofuscus*](https://www.enzolifesciences.com/BML-SE301/phospholipase-d-streptomyces-chromofuscus/), Enzo Lifesciences, cat. no. BML-SE301-0025, 250 unit / ml. |
| Reagent 8 | 5 × SDS-PAGE loading buffer: 250 mM Tris–HCl, 10% SDS (m/v), 0.5% Bromophenol blue (m/v), 50% Glycerol (v/v), 5% β-Mercaptoethanol (m/v). |
| Reagent 9 | Resolving gel (15%): 5.0 mL 30% acrylamide (29:1; Labtide, cat. no. AS-0500), 3.8 mL 1 M Tris (pH 8.8; Biosharp, cat. no. BL515B), 0.1 mL 10% SDS (Biosharp, cat. no. BL517B), 0.1 mL 10% APS (Macklin, cat.no. A801037), 4.0 μL TEMED (WEST GENE, WG2250), 3.6 g urea (Sigma-Aldrich, cat.no. U5378), supplemented with appropriate amount H_2_O to make the final volume to 10 mL. |
| Reagent 10 | Stacking gel (5%): 2.7 mL H_2_O, 0.67 mL 30% acrylamide (29:1; Labtide, cat. no. AS-0500), 0.5 mL 1 M Tris (pH 6.8; biosharp, cat. no. BL514B); 0.04 mL 10% SDS (Biosharp, cat. no. BL517B), 0.04 mL 10% APS (Macklin, cat.no. A801037); 4.0 μL TEMED (WEST GENE, WG2250). |
| Reagent 11 | 5×SDS-PAGE running buffer: 125 mM Tris; 1.25 M Glycine; 0.5% SDS (m/v). |
| Reagent 12 | Prestained protein marker: Thermo Scientific, cat. no. 26617. |
| Reagent 13 | Membrane transfer buffer: 48 mM Tris; 39 mM Glycine; 0.037% SDS, 20% methanol. |
| Reagent 14 | Hybond-C membrane with 0.22-μm (Amersham, cat. no. GE10600001). |
| Reagent 15 | 1 × TBST buffer: 20 mM Tris–HCl, 150 mM NaCl, 0.05% Tween. |
| Reagent 16 | Blocking buffer: nonfat milk (Sangon Biotech, cat. no. A600669) is dissolved with 1 × TBST buffer. |
| Reagent 17 | Anti-ATG8a antibodies: Anti-ATG8a antibodies (Abcam, cat. no. ab77003) are dissolved with 1 × TBST (1:1,000). |
| Reagent 18 | Secondary antibody: HRP-conjugated Affinipure Goat Anti-Rabbit IgG (H+L); proteintech, cat. no. SA00001-2, 1: 5,000 in 1× TBST buffer. |
| **Regents in Protocol 6** | |
| Reagent 1 | Solid MS medium: See Reagent 1 in protocol 1. |
| Reagent 2 | Liquid MS medium: See Reagent 2 in protocol 1. |
| Reagent 3 | Liquid MS–C medium: See Reagent 5 in protocol 1. |
| Reagent 4 | Liquid MS–N medium: See Reagent 6 in protocol 1. |
| Reagent 5 | Protein extraction buffer: See Reagent 5 in protocol 5. |
| Reagent 6 | 5 × SDS-PAGE loading buffer: See Reagent 8 in protocol 6. |
| Reagent 7 | Resolving gel (12%): 2.0 mL H_2_O, 4.0 mL 30% acrylamide (29:1; Labtide, cat. no. AS-0500), 3.8 mL 1 M Tris (pH 8.8; Biosharp, cat. no. BL515B), 0.1 mL 10% SDS (Biosharp, cat. no. BL517B), 0.1 mL 10% APS (Macklin, cat.no. A801037), 4.0 μL TEMED (WEST GENE, WG2250). |
| Reagent 8 | Stacking gel (5%): See Reagent 10 in protocol 5. |
| Reagent 9 | 5×SDS-PAGE running buffer: See Reagent 11 in protocol 5. |
| Reagent 10 | Prestained protein marker: See Reagent 12 in protocol 5. |
| Reagent 11 | Membrane transfer buffer: See Reagent 13 in protocol 5. |
| Reagent 12 | Hybond-C membrane with 0.45-μm (Amersham, cat. no. GE10600002). |
| Reagent 13 | 1 × TBST buffer: See Reagent 15 in protocol 5. |
| Reagent 14 | Blocking buffer: See Reagent 16 in protocol 5. |
| Reagent 15 | Anti-GFP antibodies: Anti-GFP antibodies (Abmart, cat. no. M20004) are diluted with 1×TBST (1:3000). |
| Reagent 16 | Secondary antibody: HRP-conjugated Affinipure Goat Anti-Mouse IgG (H+L); proteintech, cat. no. SA00001-1, 1: 5,000 in 1× TBST buffer. |
